# Anterolateral Miniopen Triple‐Row Repair With Acellular Human Dermal Matrix Patch Augmentation for Larger Rotator Cuff Tears

**DOI:** 10.1002/atn2.70266

**Published:** 2026-09-28

**Authors:** Chul‐Hyun Cho, Byung‐Chan Choi, Hyuk‐Joon Sohn, Du‐Han Kim

**Affiliations:** ^1^ Department of Orthopedic Surgery Keimyung University Dongsan Hospital Keimyung University School of Medicine Daegu South Korea

## Abstract

Large to massive rotator cuff tears are frequently associated with poor tendon quality and high rates of structural failure after repair. Acellular human dermal matrix patch augmentation combined with rotator cuff repair has been introduced to enhance bone‐to‐tendon healing by providing early postoperative mechanical strength. However, fully arthroscopic patch augmentation techniques are technically demanding and associated with increased operative complexity. The described technique combines arthroscopic evaluation and subacromial decompression with an anterolateral miniopen triple‐row repair and onlay acellular human dermal matrix patch augmentation. This approach simplifies patch handling while providing strong fixation and may represent a practical alternative to fully arthroscopic patch augmentation techniques.

## Graphical abstract

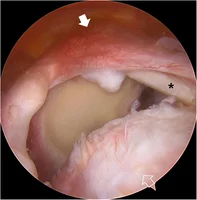

**VIDEO 1:** Step‐by‐step presentation of an anterolateral miniopen triple‐row rotator cuff repair with acellular human dermal matrix patch augmentation. Video content can be viewed at https://doi.org/10.1002/atn2.70266.

Rotator cuff repair (RCR) is a commonly performed procedure in orthopaedic fields.^1^, ^2^ Despite advances in surgical techniques and instrumentation, high retear rates after RCR have been reported, particularly in patients with large to massive rotator cuff tears (RCTs).^3^, ^4^ Although structural failure does not always correlate with poor clinical outcomes, several studies have shown that intact tendon healing is generally associated with superior long‐term results.^5^, ^6^

Patch augmentation using acellular human dermal matrix (AHDM) has been introduced to reinforce RCR by improving load sharing, increasing repair strength, and providing a biologic scaffold for tendon healing.^6^, ^7^, ^8^, ^9^ Previous studies have reported improved structural integrity and reduced retear rates in patients with large or massive RCTs.^10^, ^11^, ^12^, ^13^ However, most full arthroscopic techniques are technically demanding, requiring advanced arthroscopic skills and prolonged operative time with intraoperative complexity.

Miniopen RCR remains a valuable option for large tears, offering direct visualization, reliable tendon mobilization, and secure suture management, while minimizing deltoid morbidity when performed through a limited incision.^14^, ^15^, ^16^ This Technical Note describes a practical and reproducible anterolateral miniopen triple‐row RCR augmented with an AHDM patch for the treatment of large to massive RCTs. A detailed step‐by‐step description and accompanying video are provided.

## SURGICAL TECHNIQUE

The complete surgical technique is shown in Video 1.

### Diagnostic Arthroscopy and Subacromial Decompression

Under general anesthesia, the patient is placed in the lateral decubitus position with a posterior tilt of 20° to 30°. Standard diagnostic arthroscopy of the glenohumeral joint is performed using anterior and posterior portals. The arthroscope is then advanced into the subacromial space through the posterior portal, and a lateral portal is established.

Using a probe, the size, configuration, and mobility of the RCT are carefully assessed. Careful debridement of the margin of the torn rotator cuff and adhesiolysis are performed to improve tendon mobility (Figure 1). Subacromial spurs and acromial wear are evaluated, and arthroscopic acromioplasty is performed using a burr when signs of impingement are present.

**FIGURE 1:**
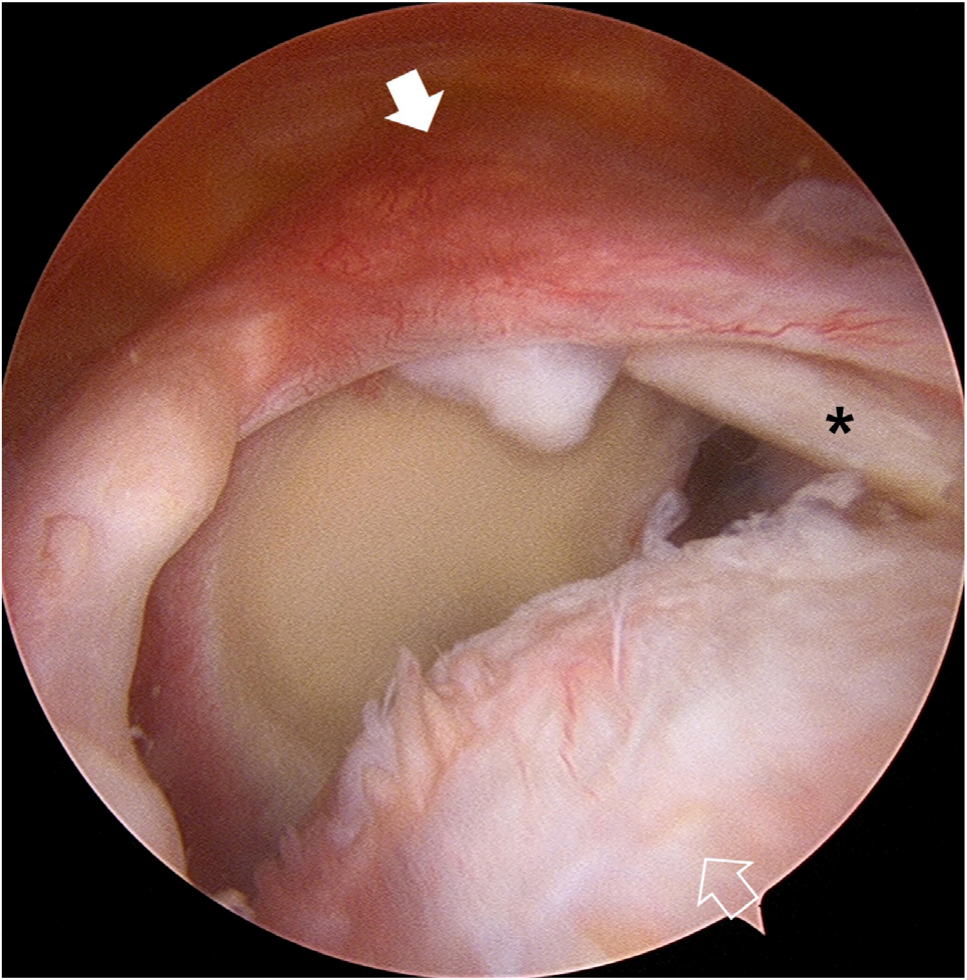
Arthroscopic view from the lateral viewing portal showing a massive rotator cuff tear in the right shoulder (full arrow, torn rotator cuff; empty arrow, greater tuberosity; *, biceps tension).

### Anterolateral Miniopen RCR

Miniopen RCR is performed via an anterolateral approach as previously described.^14^, ^15^ A 3‐ to 4‐cm skin incision is made from the anterolateral edge of the acromion distally. The raphe between the anterior and middle deltoid is carefully dissected, and adequate exposure is obtained using a deltoid retractor. The subacromial bursa is excised to expose the torn rotator cuff.

Traction sutures are placed in the torn tendons, and the tear configuration and tendon involvement are confirmed by rotating the arm. After complete tendon mobilization, reduction to the footprint of the greater tuberosity is attempted. Then, footprint preparation is performed using the crimson duvet procedure.

Two medial‐row suture anchors (4.75 mm HEALICOIL, Smith & Nephew, Andover, MA) are placed at the articular margin of the greater tuberosity. Sutures are passed through the medial portion of the torn tendons at a depth of approximately 10 to 12 mm. One or 2 intermediate‐row suture anchors (2.8 mm Y‐Knot, CONMED, Utica, NY) are then inserted into the lateral aspect of the greater tuberosity. Sutures are passed through the tendon and secured using a sliding knot‐tying technique (Figure 2).

**FIGURE 2:**
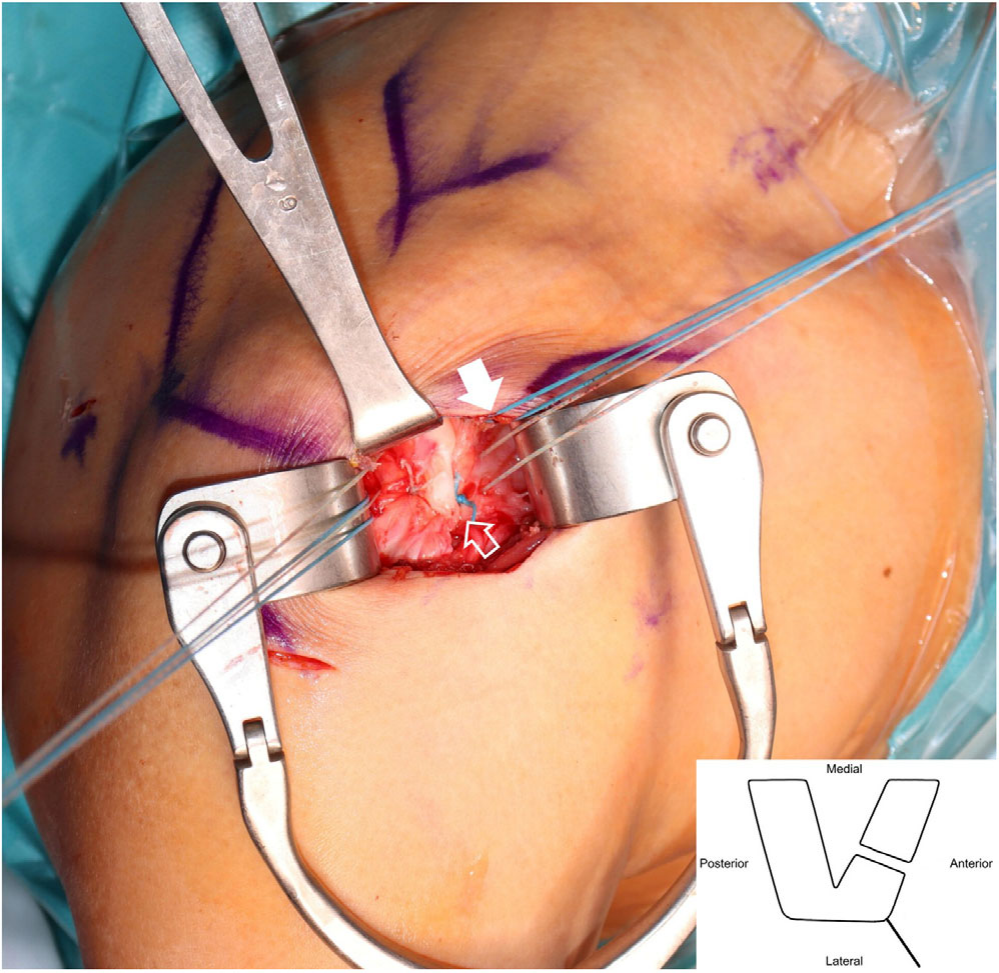
Right shoulder, lateral decubitus position, posterosuperior viewing portal. After the miniopen approach, medial‐row suturing (full arrow) and intermediate‐row fixation (empty arrow) of the torn rotator cuff tendon.

### AHDM Patch Augmentation

An AHDM patch (3‐mm thickness, 3 × 4 cm; ConPatch, MSBIO, Seongnam, Republic of Korea) is applied in an onlay fashion to reinforce the repair. Medial‐row suture limbs are passed through the patch (Figure 3) and tied using a sliding knot‐tying technique. These sutures are then secured laterally using knotless anchors (Kinex Fix2Lock; OSTEONIC, Seoul, Republic of Korea) (Figure 4). A schematic illustration and magnetic resonance imaging of the triple‐row repair with AHDM patch augmentation are shown in Figures 5 and 6.

**FIGURE 3:**
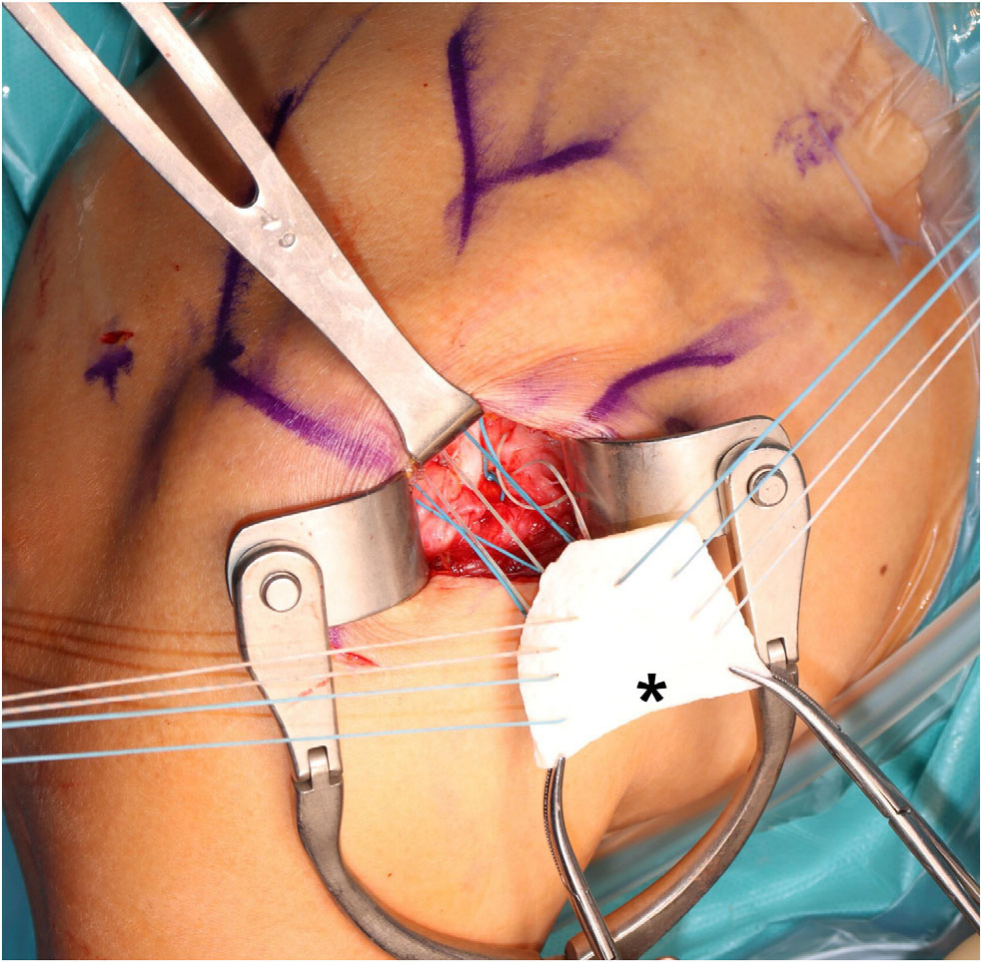
Right shoulder, lateral decubitus position, posterosuperior viewing portal. Passing of medial‐row sutures through the acellular human dermal matrix patch (*).

**FIGURE 4:**
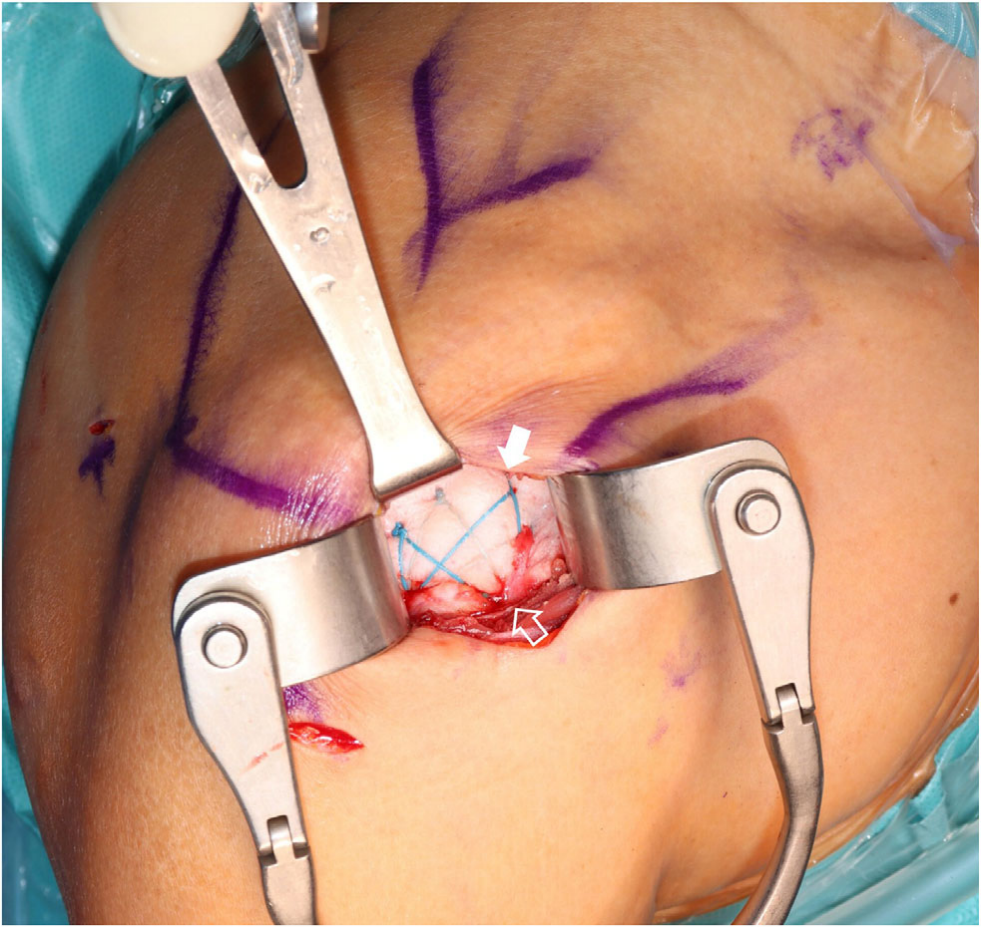
Right shoulder, lateral decubitus position, posterosuperior viewing portal. Lateral‐row fixation (empty arrow) after medial knot tying (full arrow) with the application of acellular human dermal matrix patch.

**FIGURE 5:**
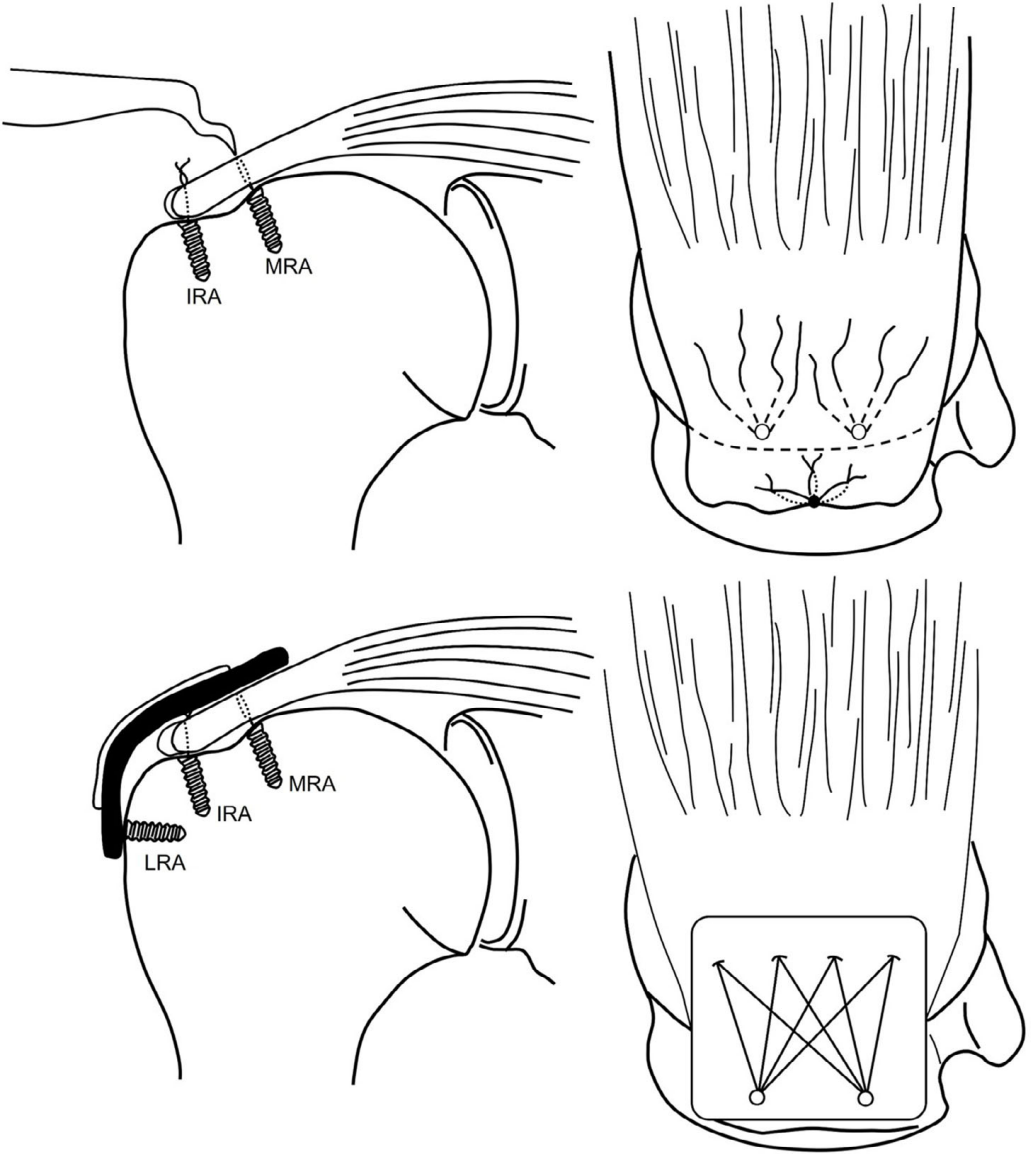
Schematic illustrations of triple‐row repair with patch augmentation. (IRA, intermediate row anchor; LRA, lateral row anchor; MRA, medial row anchor.)

**FIGURE 6:**
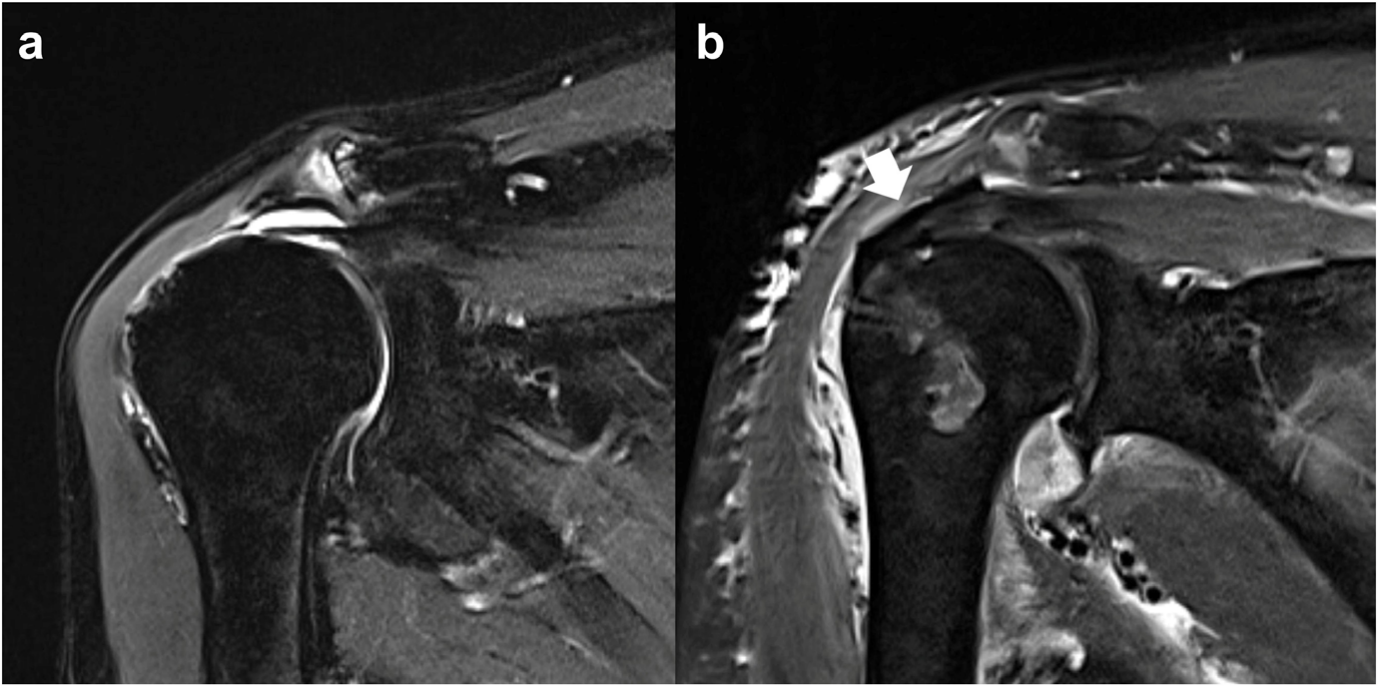
(a) Preoperative and (b) postoperative magnetic resonance imaging of the right shoulder showing patch augmentation combined with rotator cuff repair (full arrow).

To prevent deltoid detachment, a No. 1 absorbable suture is passed through a bone tunnel created in the acromion for secure deltoid reattachment. Additional sutures are placed 3 to 4 times to repair the anterior and middle deltoid.

### Postoperative Rehabilitation

Patients are immobilized in an abduction brace for 6 weeks postoperatively. Passive range‐of‐motion exercises are initiated at 4 to 6 weeks after surgery depending on tear size and repair quality. Active range‐of‐motion exercises begin at 8 weeks, followed by strengthening exercises at 3 months. Return to sports or occupational activities is permitted approximately 6 months after surgery.

### Ethical Approval

Institutional Review Board approval was waived because this article describes a surgical technique without patient‐specific data. Informed consent was obtained from the patient for use of images and video.

## DISCUSSION

The key finding of this Technical Note is that an anterolateral miniopen triple‐row RCR with AHDM patch augmentation provides strong fixation while simplifying patch handling and suture management. This technique may serve as a practical and reproducible alternative to fully arthroscopic patch augmentation.

This approach may be particularly beneficial in patients with large or massive RCTs, which are associated with high failure rates because of excessive repair tension and poor tendon quality. Triple‐row constructs incorporating medial, intermediate, and lateral fixation with onlay patch augmentation distribute load more evenly across the footprint and reduce gap formation compared with nonaugmented single‐ or double‐row repairs. In addition, AHDM patch augmentation may enhance early postoperative repair strength and promote tendon healing by serving as a biologic scaffold for cellular ingrowth.

Compared with fully arthroscopic techniques, the miniopen approach offers several advantages. Direct visualization facilitates precise tendon mobilization, accurate footprint preparation, and secure suture handling, particularly in retracted or delaminated tears (Table 1). Patch handling and fixation are simplified, potentially reducing operative time and technical complexity (Table 2).

**TABLE 1:** Indications and Contraindications

**Indications**	**Contraindications**
Large or massive rotator cuff tears (>3 cm)	Irreparable rotator cuff tears
Poor tendon quality requiring reinforcement	Advanced fatty infiltration of rotator cuff muscles (Goutallier grade 3 or 4)
Retracted tears requiring extensive mobilization	Moderate to severe glenohumeral arthritis
Revision rotator cuff repair	Suspicious infection
Patients with high functional demand	Deltoid dysfunction or prior deltoid detachment
Incomplete footprint coverage (>50%) after conventional repair	Inability to comply with postoperative rehabilitation

**TABLE 2:** Advantages and Disadvantages

**Advantages**	**Disadvantages**
Strong biomechanical fixation with improved footprint compression	More invasive than fully arthroscopic techniques
AHDM patch augmentation enhances repair strength and biologic healing	Increased cost because of graft and implants
Direct visualization facilitates tendon mobilization and footprint preparation	Risk of deltoid injury if not properly handled
Simplified graft handling compared with arthroscopic patch techniques	Requires familiarity with both arthroscopic and open techniques
Particularly useful for large or poor‐quality rotator cuff tears	Limited applicability in irreparable tears or advanced fatty degeneration

AHDM, acellular human dermal matrix.

Nevertheless, several limitations should be considered. Visualization may be limited in obese or muscular patients, and there is a potential risk of deltoid injury if the procedure is not performed carefully (Table 3). However, deltoid integrity can be preserved through meticulous splitting along the deltoid raphe and secure reattachment.

**TABLE 3:** Pearls and Pitfalls

**Pearls**	**Pitfalls**
Perform thorough diagnostic arthroscopy to assess tear configuration and reparability	Inadequate tear mobilization leading to excessive repair tension
Preserve the deltoid by splitting along the raphe between anterior and middle deltoid	Excessive deltoid detachment or insufficient reattachment to the acromion
If necessary (i.e., in cases with limited cuff mobility), the surgeon can medialize the footprint 3‐5 mm to reduce tension on the repair	Inadequate footprint preparation compromising tendon healing
Achieve complete footprint preparation using the crimson duvet technique	
Use traction sutures to facilitate tendon mobilization and orientation	Malpositioned sutures causing tendon strangulation
Apply the AHDM patch in an onlay fashion after completion of tendon repair	Overtensioning the patch, which may restrict tendon motion
Secure medial‐row sutures through both tendon and patch for reinforcement	
Confirm stable fixation through full passive range of motion before closure	

AHDM, acellular human dermal matrix.

Anterolateral miniopen triple‐row RCR with AHDM patch augmentation is a practical and reproducible technique for the treatment of large to massive RCTs. This approach may represent a valuable alternative to fully arthroscopic patch‐augmented repairs in appropriately selected patients.

## DISCLOSURES

The authors (C‐H.C., B‐C.C., H‐J.S., D‐H.K.) declare that they have no known competing financial interests or personal relationships that could have appeared to influence the work reported in this article.
